# Atypical Firearm Injury to the Anterior Triangle of the Neck With an Unusual Projectile Trajectory: A Rare Case Report

**DOI:** 10.7759/cureus.33875

**Published:** 2023-01-17

**Authors:** Anil Kumar, Harendra Kumar, Anurag Kumar, Majid Anwer, Deepak Kumar

**Affiliations:** 1 Trauma and Emergency, All India Institute of Medical Sciences, Patna, Patna, IND; 2 Trauma Surgery, All India Institute of Medical Sciences, Patna, Patna, IND; 3 General Surgery, All India Institute of Medical Sciences, Patna, Patna, IND

**Keywords:** anterior triangle, gunshot injuries, bullet trajectory, wound ballistics, neck

## Abstract

The use of firearms is increasing in our society, which increases the incidence of gunshot injuries in developing countries like India. Gunshot injuries in the neck regions are significantly associated with high mortality and morbidity because of the major vascular and other vital structures present in the neck. However, it’s very rare that a bullet may have a trajectory that passes through the neck region and does not damage the vital structures. We present one such case of gunshot injury to the neck. A 20-year-old male reported to the emergency department after sustaining a gunshot wound to the left anterior cervical region of the neck. On examination, the right sternocleidomastoid muscle was taut and tender to the touch. It is very rare that a bullet injury in the neck without damage to even a single vital structure. The most critical steps in managing patients with high-velocity penetrating injuries to the head and neck region are securing an airway, controlling hemorrhage, and identifying and repairing residual traumatic deformities at the earliest possible key points for the best outcome.

## Introduction

Gunshot injuries in the neck cause profound morbidity and significant mortality. Gunshot injuries are mainly homicidal in nature, followed by suicide and accidental [1]. In the last decade, firearm injuries have increased worldwide, especially in developing countries like India [2]. Because of the complex anatomy and very close location of all vital structures present in the neck, neck injuries are more likely to be fatal [3]. Any delay in the diagnosis and treatment of these vital organs or injuries can have devastating consequences [4]. Therefore, appropriate management supported by a relevant understanding of wound ballistics should be provided to gunshot wound victims. The survival of a patient with a gunshot transversely entering the entire neck across its deep tissues is unknown. In spite of the bullet following a nonlinear trajectory, no damage to the deeper vital structures was evident. By examining the case from several angles, we examine the clinical implications of wound ballistics in gunshot injuries to evaluate the mechanisms driving the unpredictable nonlinear bullet trajectory. This research has been reported in line with surgical case report (SCARE) [5].

## Case presentation

**Figure 1:**
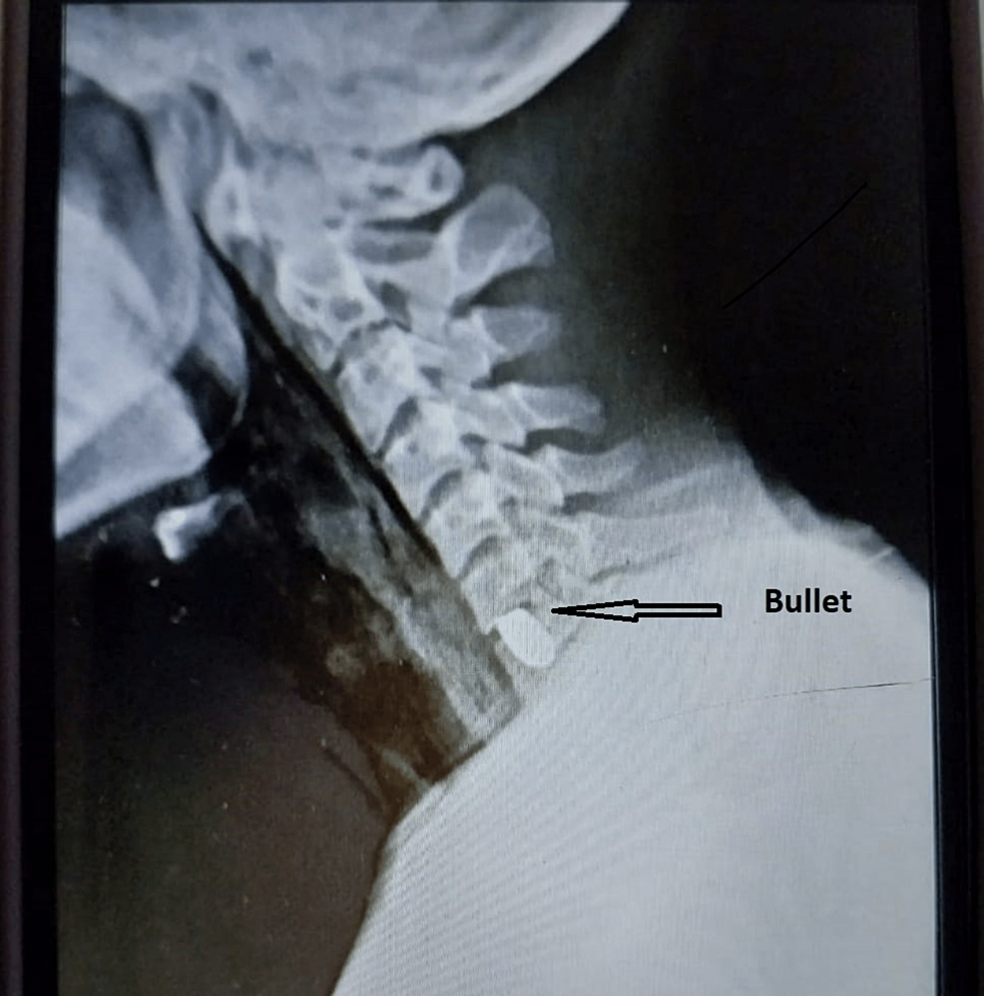
Bullet marked with arrow shows bullet in a lateral x-ray film of the neck.

**Figure 2:**
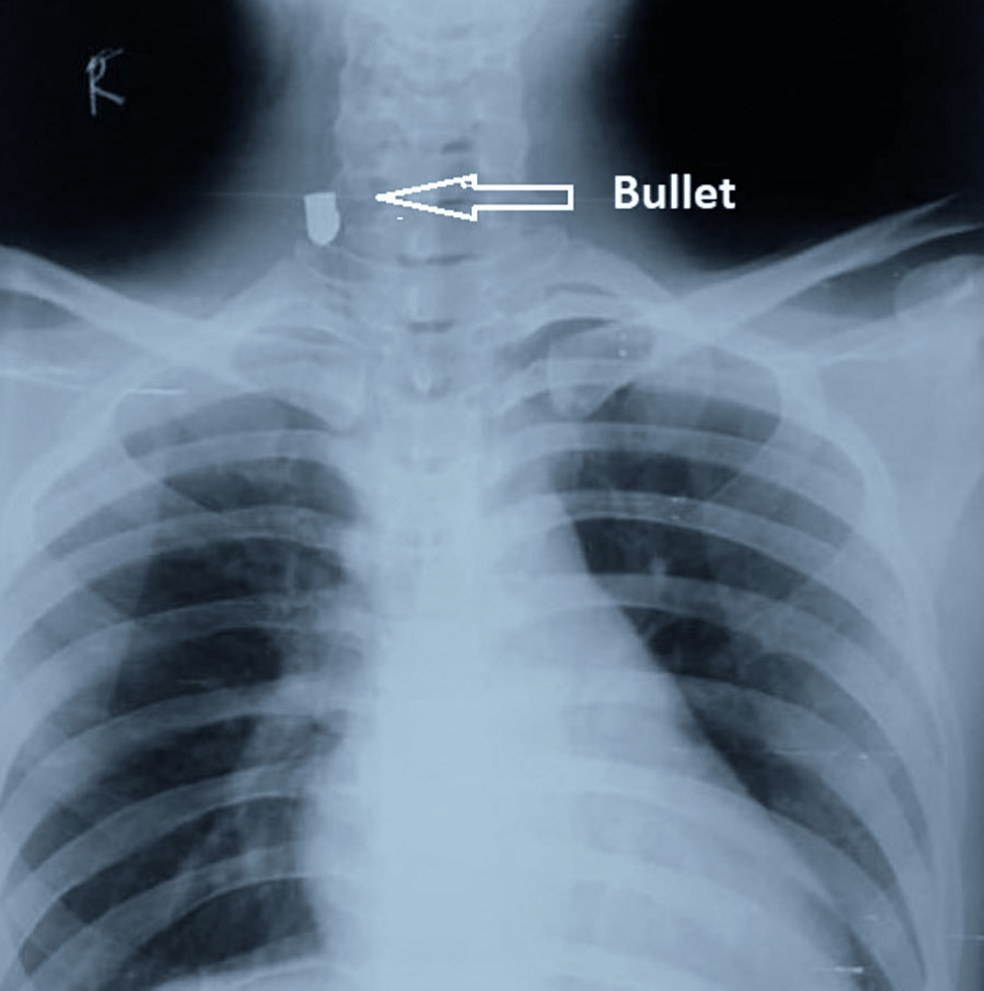
Bullet at C6-C7 level in antero-posterior view of x-ray neck and chest.

A 20-year-old male presented to the emergency department (ED) with a single gunshot wound over the left side of the neck in zone 2 in the submandibular area. He was brought by police after 14 hours of gunshot injury. His presenting complaint was neck pain and anxiety. On examination, the right sternocleidomastoid muscle was taut and tender to the touch. The vital signs were within normal limits, and neurological abnormalities, including sensory evaluation, were unaltered for pinprick and mild touch throughout. All laboratory investigations were within normal limits. On the primary survey, his airway was patent, breathing was spontaneous, respiratory rate was 18 per minute and SpO_2_ was 100%, pulse was 84 per minute, blood pressure was 110/70 mmHg, and Glasgow Coma Scale (GCS) was E4V5M6. The bullet entry wound was found in the left submandibular region of the neck. Anywhere else on the head and neck, the bullet's potential exit wound could not be found. There was no carotid bruits. X-ray of the neck showed a bullet in between C6 and C7 (Figures 1, 2). His blood sample was sent for grouping and cross-matching to arrange four units of blood product.

**Figure 3:**
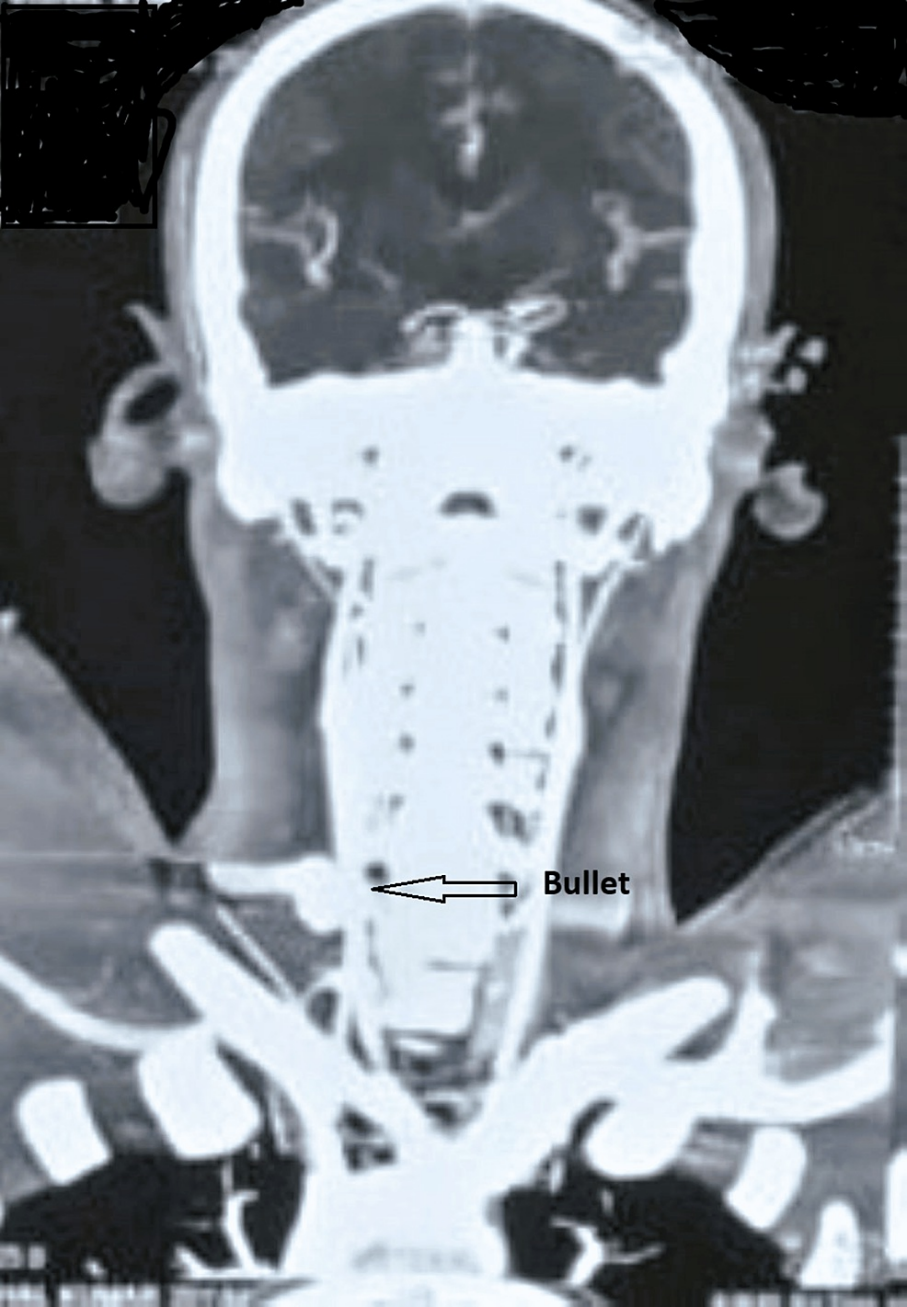
CT-angiography shows no contrast leak and a bullet marked with an arrow. CT: computed tomography.

Initial computed tomography (CT) scan (Figure 3) and ultrasound neck revealed a bullet fragment on the right side of the neck between levels C6 and C7.

**Figure 4:**
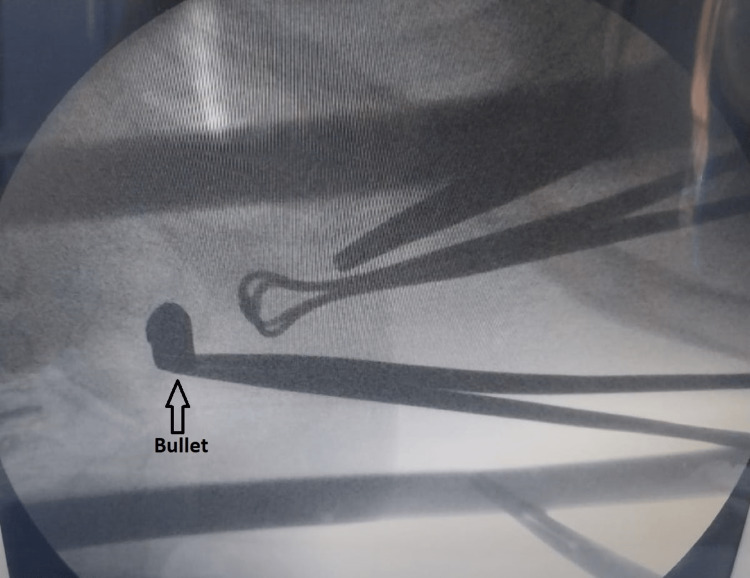
Intra-operative C-arm view of bullet marked with an arrow.

While dynamic CT revealed no signs of active extravasations, CT also revealed soft tissue edema and small air sacs among deep tissue structures, such as fat, muscles, fascia, and connective tissues, seemingly following the bullet route. On examination, the bullet was not felt. In view to consider the possible risk of complications like pseudoaneurysm and embolism, our priority was to remove the bullet. Under general anesthesia, the surgical site was prepared, marked, and surgical exploration through the right vertical incision on the right anterior cervical region was carried out utilizing a C-arm (x-ray image intensifier) to find the bullet (Figure 4). The surgery took three hours to retrieve the bullet. The barium meal and bronchoscopy were done on the third post-operative day with normal findings. The post-operative period was uneventful, and the patient was discharged on the eighth post-operative day. The patient is on regular follow-up, and he is doing well.

**Figure 5:**
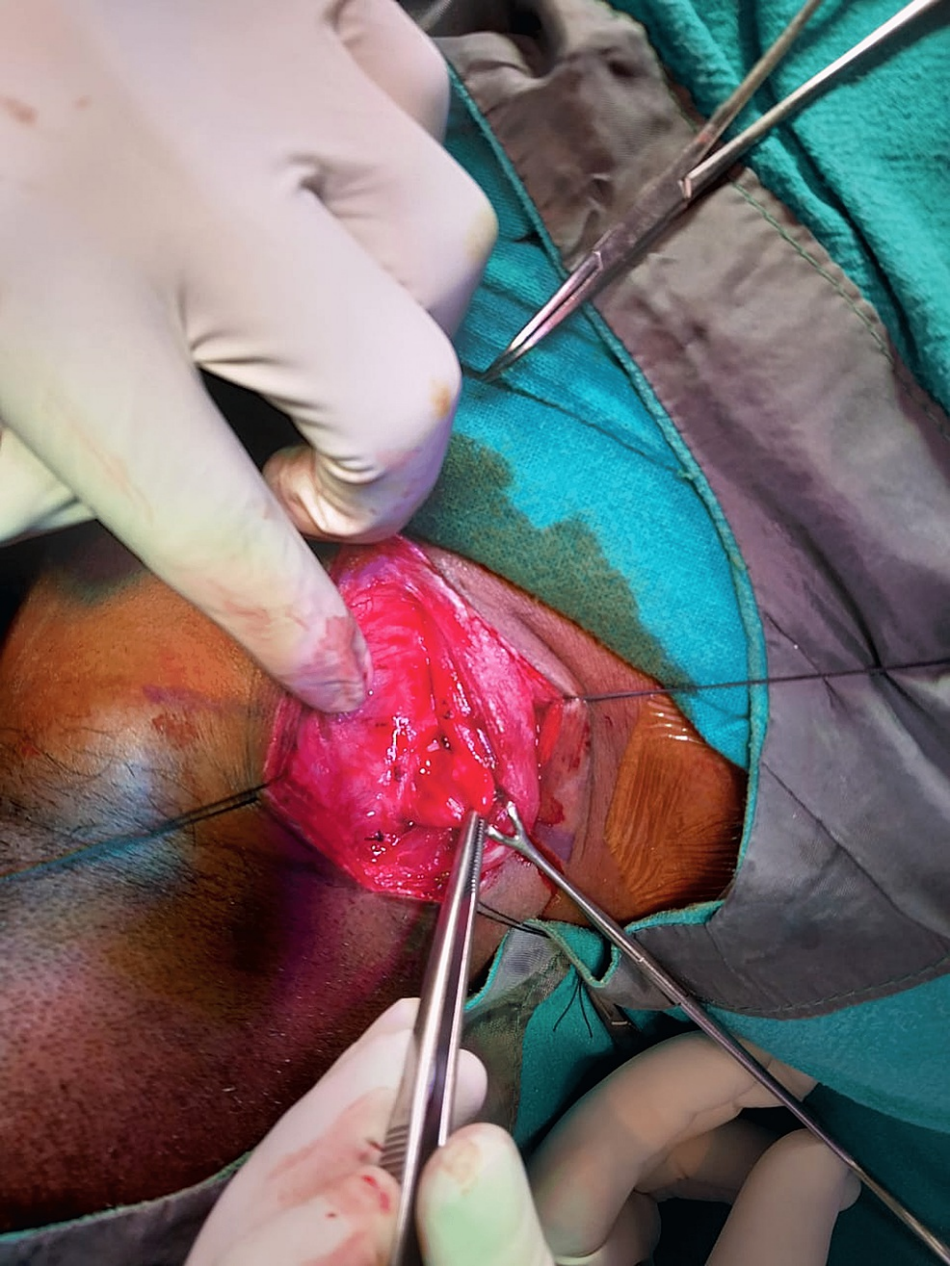
Intra-operative picture of bullet removal.

**Figure 6:**
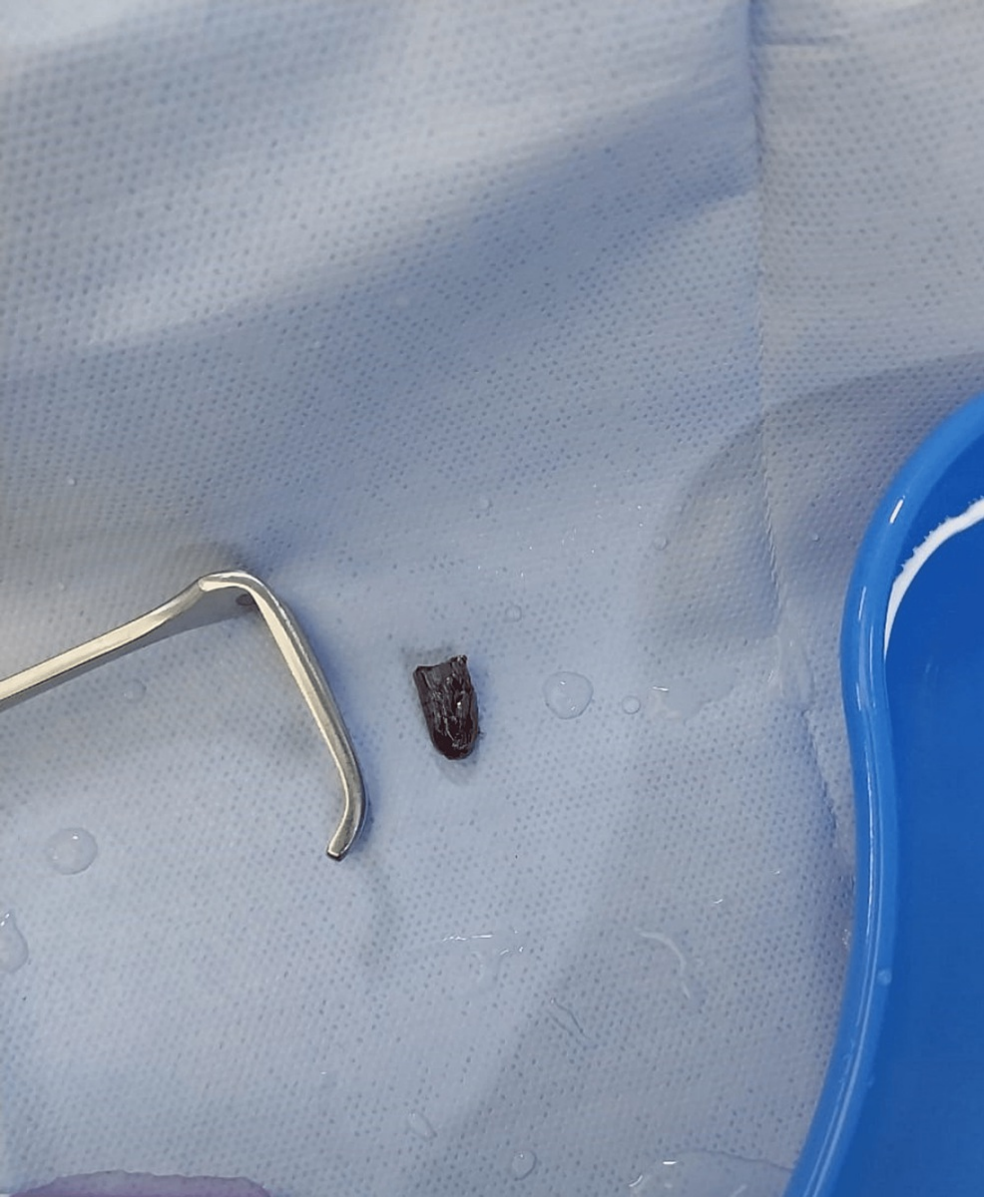
Extracted bullet.

The bullet was located and taken out (Figures 5, 6).

## Discussion

The incidence of gunshot injuries is increasing day by day because of multiple factors, including increasing population, unemployment, lack of education, increase in terrorism, ego clash, and decrease in the tolerance level of people [2]. The penetrating neck injuries may be fatal because major vessels, including carotid, subclavian, and vertebral arteries, are present in neck zones [6-8]. In penetrating neck injuries, the cause of delayed death and morbidity is mainly due to pharyngoesophageal injuries [9]. Because the head and neck regions contain a lot of critical structures in a confined space, even the minimal motion of a penetrating bullet can simultaneously cause severe significant vascular and nerve injuries resulting in serious life-threatening conditions. An immediate surgical exploration may be required in the case of a pulsatile hematoma, active bleeding, airway compromise, large subcutaneous hematoma, and shock [10]. This physical characteristic is evident in the high death rates for head- and neck-related gunshot wounds, which can range from 35% to 36% [11,12]. The head and neck region accounts for only 13.8%-20% of all gunshot injuries [11-13], but it causes 54%-58% of all firearm-related deaths [11,12]. The most common zone of neck injury in penetrating trauma is zone II [14], as in the present case. According to a study done by Mahmoodie et al., the most common cause of penetrating neck trauma was stab injuries, while the study by Alao suggested causes were physical assault, firearm injuries, and stab injuries [15,16]. The unusual trajectory of the bullet in the chest wall was also reported in the literature [17], like the index case. In such cases, the exact path of the bullet can be determined by radiological investigations, preferably contrast-enhanced computed tomography (CECT). The prediction of the path is more challenging in the case of high-velocity projectile wounding because the bullet may follow a complicated path rather than a linear path. In cases of an absent exit wound, the entry wound should be carefully explored under the guidance of a C-arm, as in the present case. The prognosis of a gunshot injury in the neck depends upon the damage to neurovascular structures and the multidisciplinary approach. In the present case, it is by chance that even after the trajectory of the bullet went from zone II to zone I, the patient did not suffer a life-threatening injury. We need to recommend guidelines to prevent the incidence of gunshot injuries and their consequences by limiting the number of handgun purchases, regulations to check the illegal trade of firearms, accountability of ammunition used, and developing standardized health care [2].

## Conclusions

The most important steps in managing penetrating head and neck injuries are securing an airway, controlling hemorrhage, identifying other injuries, and repairing of residual traumatic deformities. By understanding the wound ballistics, one can anticipate the magnitude of tissue injury, detect any latent damage to internal tissues, and estimate and interpret the bullet trajectory and the damage caused. In cases with an atypical track, this is of greater significance. Optimal management of gunshot injuries is hence dependent on these factors.
